# Supplementary figures and images for: Time to major adverse drug reactions and its predictors among children on antiretroviral treatment at northwest Amhara selected public hospitals northwest; Ethiopia, 2023
Source: PLoS One. 2024 Oct 3;19(10):e0309796. doi: 10.1371/journal.pone.0309796 (PMC11449323; doi:10.1371/journal.pone.0309796)

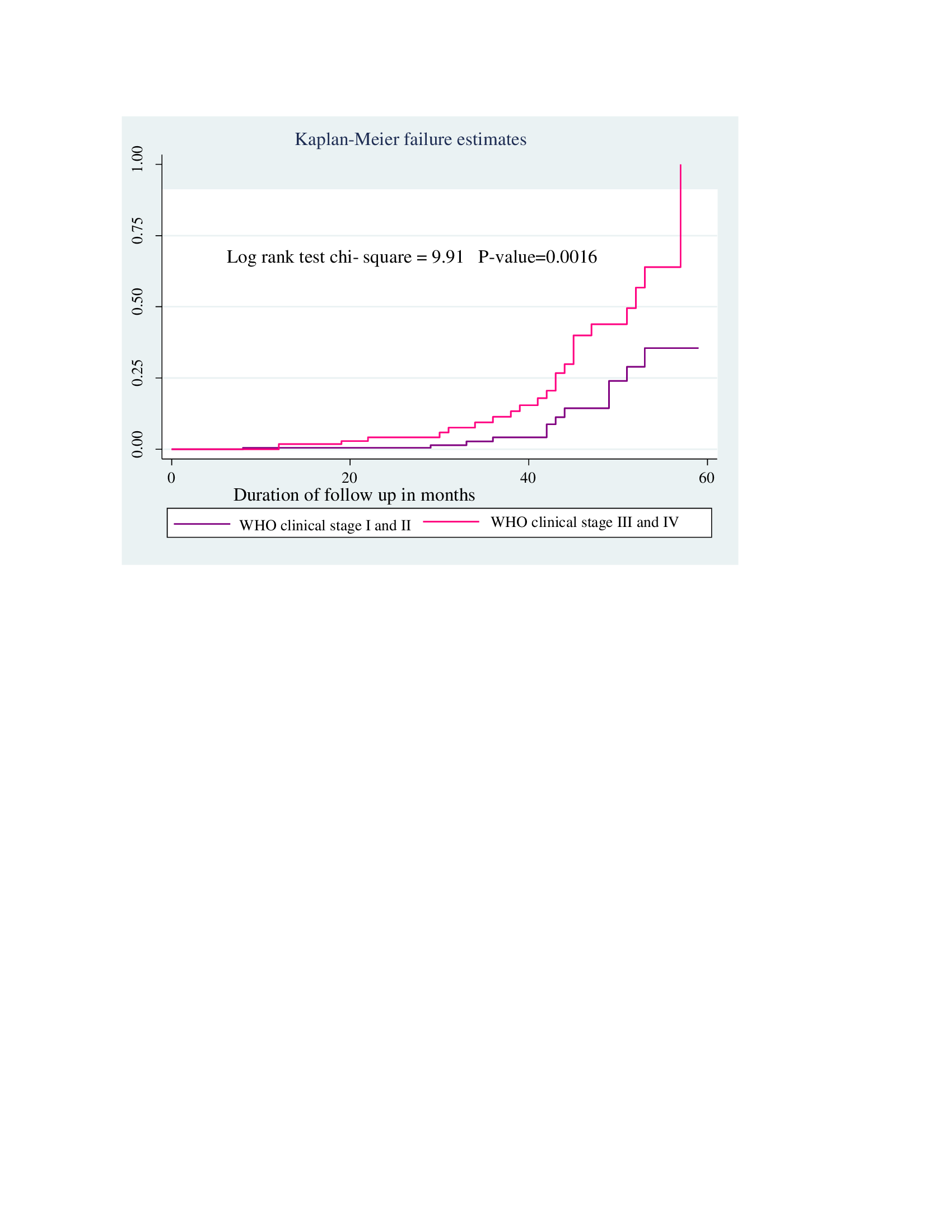

Supplement: S1 Fig — (n = 380). (TIF) [file pone.0309796.s002.tif]

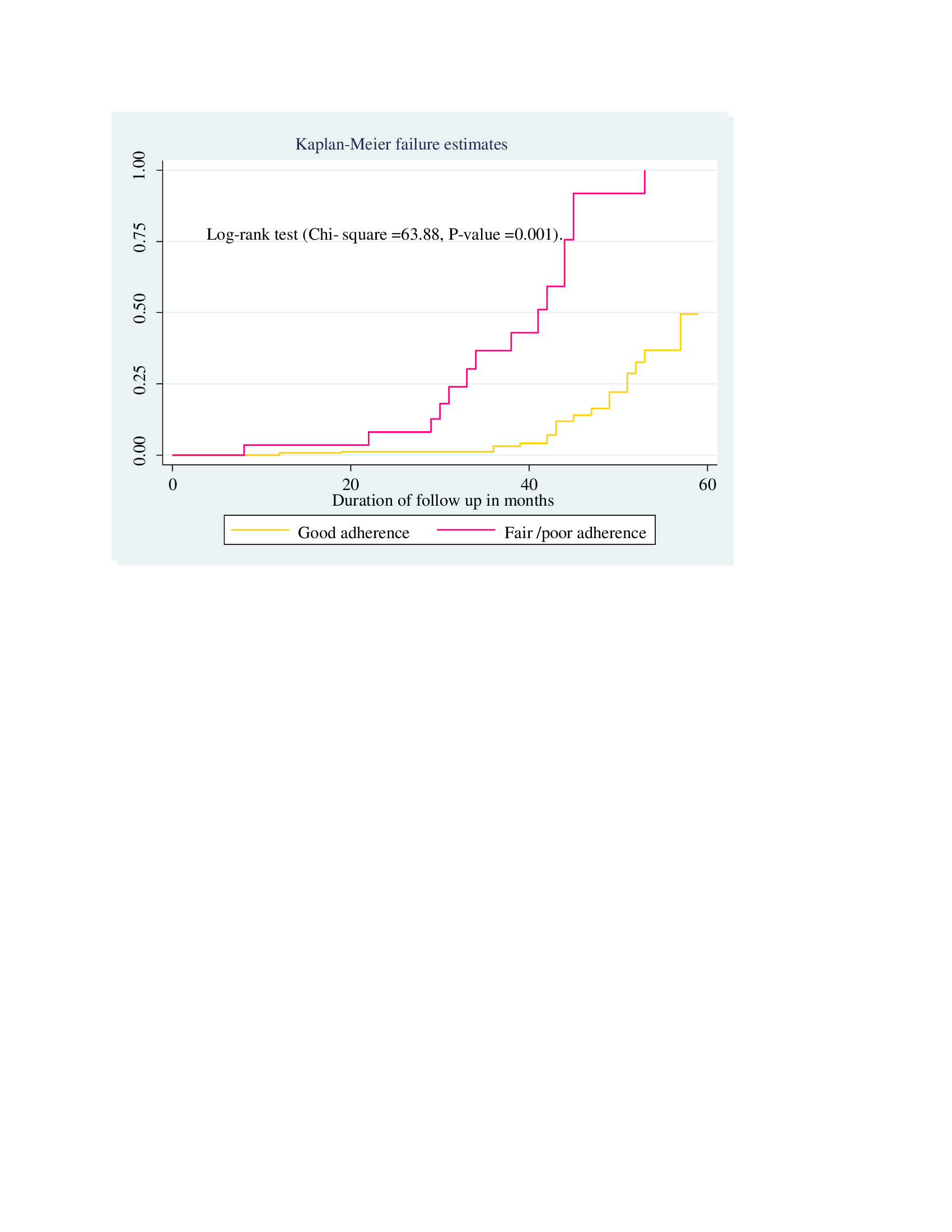

Supplement: S2 Fig — (n = 380). (TIF) [file pone.0309796.s003.tif]

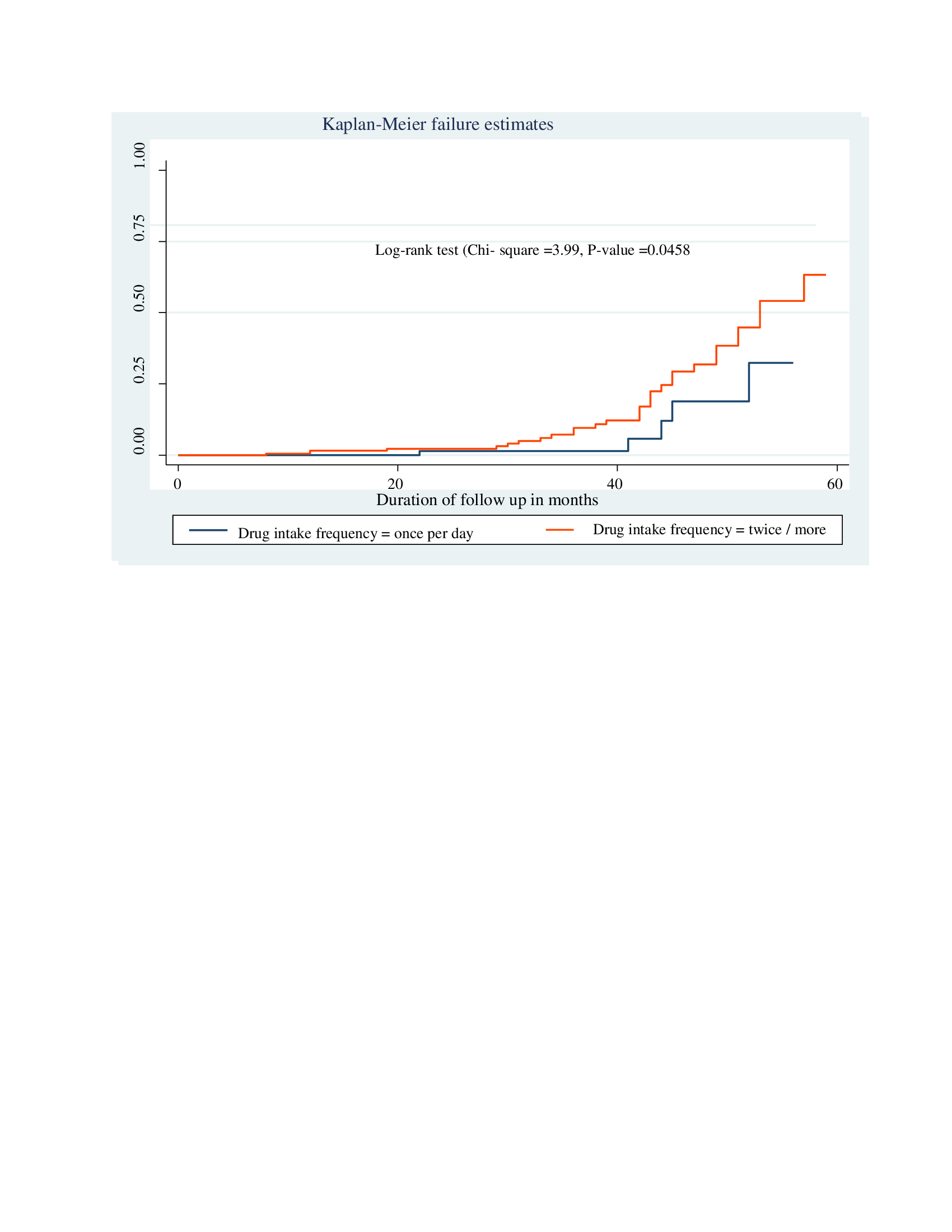

Supplement: S3 Fig — (n = 380). (TIF) [file pone.0309796.s004.tif]

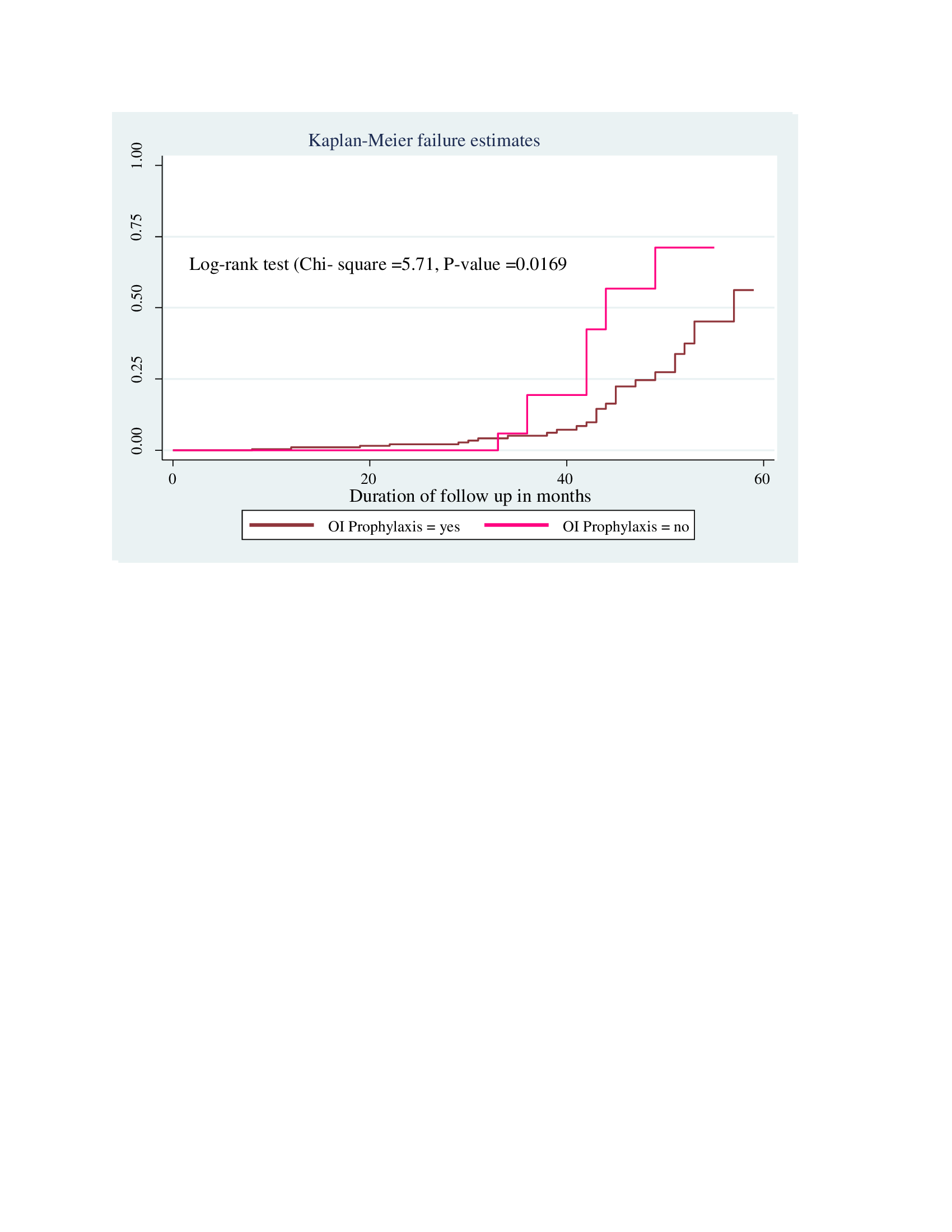

Supplement: S4 Fig — (n = 380). (TIF) [file pone.0309796.s005.tif]
