# Supplementary material for: Time to major adverse drug reactions and its predictors among children on antiretroviral treatment at northwest Amhara selected public hospitals northwest; Ethiopia, 2023
Source: PLoS One. 2024 Oct 3;19(10):e0309796. doi: 10.1371/journal.pone.0309796 (PMC11449323; doi:10.1371/journal.pone.0309796)
Supplement: S1 Table — (n = 380). (DOCX) [file pone.0309796.s006.docx]

**S1 Table 1:** Life table for MADRs survival among children on ART at selected public health selected public hospital, Northwest Amhara, Ethiopia , 2023.(n=380)

| Time interval in months | Number  Entering  Interval | Number Withdrawing  during Interval | Number of Risk | Patients experience MADRs | Probability of MADRs | | Probability of  Surviving | Cumulative Probability  Surviving at end of  Interval | 95%CI |
| --- | --- | --- | --- | --- | --- | --- | --- | --- | --- |
| 0-12 | 380 | 70 | 345 | 3 | | 0.00869 | 0.9913 | 0.9913 | 0.97,0.99 |
| 12-24 | 307 | 122 | 246 | 2 | | 0.00813 | 0.9918 | 0.9831 | 0.95,0.99 |
| 24-36 | 183 | 70 | 148 | 7 | | 0.0473 | 0.9527 | 0.9365 | 0.87,0.96 |
| 36-48 | 106 | 33 | 89 | 15 | | 0.1685 | 0.8314 | 0.7786 | 0.59,0.80 |
| 48-60 | 57 | 1 | 56 | 8 | | 0.1428 | 0.8571 | 0.6673 | 0.20,0.61 |
