# Supplementary material for: Time to major adverse drug reactions and its predictors among children on antiretroviral treatment at northwest Amhara selected public hospitals northwest; Ethiopia, 2023
Source: PLoS One. 2024 Oct 3;19(10):e0309796. doi: 10.1371/journal.pone.0309796 (PMC11449323; doi:10.1371/journal.pone.0309796)
Supplement: S2 Table — (n = 380). (DOCX) [file pone.0309796.s007.docx]

**S1 Table 2:** Global test result of variables among HIV positive children on ART, at selected public hospital Northwest Amhara, Ethiopia, 2023. (n=380)

| Variable | Df. | Chi-square | P-value |
| --- | --- | --- | --- |
| OI infection | 1 | 1.57 | 0.2106 |
| Current status of patient | 1 | 0.43 | 0.5098 |
| Prophylaxis provided | 1 | 3.30 | 0.0694 |
| INH prophylaxis | 1 | 1.26 | 0.2610 |
| WHO Clinical Stage | 1 | 0.16 | 0.6925 |
| ART drug intake frequency | 1 | 0.01 | 0.9321 |
| Adherence to ART | 1 | 0.27 | 0.600 |
