# Supplementary material for: Time to major adverse drug reactions and its predictors among children on antiretroviral treatment at northwest Amhara selected public hospitals northwest; Ethiopia, 2023
Source: PLoS One. 2024 Oct 3;19(10):e0309796. doi: 10.1371/journal.pone.0309796 (PMC11449323; doi:10.1371/journal.pone.0309796)
Supplement: S3 Table — (n = 380). (DOCX) [file pone.0309796.s008.docx]

**S1 Table 3:** Parametric and semi parametric model comparison among HIV positive children on ART, at selected public hospital Northwest Amhara, Ethiopia, 2023. (n=380)

|  | Cox PH | Log logistic | Exponential | Gompertz | Log norm | Weibull |
| --- | --- | --- | --- | --- | --- | --- |
| Log likelihood | -118.11701 | -37.930432 | -79.266289 | -31.354711 | -40.390816 | -36.895642 |
| AIC | 252.234 | 93.86086 | 174.5326 | 80.70942 | 98.78163 | 91.79128 |
| BIC | 283.7554 | 129.3224 | 206.0539 | 116.171 | 134.2432 | 127.2528 |
