# Supplementary material for: Time to major adverse drug reactions and its predictors among children on antiretroviral treatment at northwest Amhara selected public hospitals northwest; Ethiopia, 2023
Source: PLoS One. 2024 Oct 3;19(10):e0309796. doi: 10.1371/journal.pone.0309796 (PMC11449323; doi:10.1371/journal.pone.0309796)
Supplement: Attachment — Submitted filename: R_enyn PONE -D-23-20912 R1.docx [file pone.0309796.s009.docx]

**Opening Comments:**

This is an important by Bantegizie Senay and colleagues. It sought to retrospectively examine adherence of children infected with HIV to ARTs’ in Amhara in Ethiopia.

The manuscript is well written...

**Title**:

Time to Major Adverse Drug Reactions and Its Predictors Among Children on Antiretroviral Treatment at Northwest Amhara Selected Public Hospitals Northwest; Ethiopia, 2023.

**Abstract**

- The abstract is ok, and well structured.

**Introduction**

- Paragraph 5, line 5: Revise ‘4.18–2.3 gm/dl’ to read **4.2 -2.3 g/dl**
- Paragraph 6, line 2: Revise ….’majority of (90.74%)’….to read ….’majority (90.7%) of’ …., and delete the last 2 words (i.e. of ART ) at the end of that sentence.
- Last Paragraph, second line: Write the acronym ‘MADRs’ in full, and put the MADRs in parentheses or bracket here

**Methods**

***Study area, setting, and population***

- Revise the first sentence into 2 sentences

- Line 6 and 7, add the respective hospitals to complete that sentence

**Data Quality Control**

- Last sentence, from Line 4: Should read 5% with the number obtained in parentheses

- Write DMCSH and FHCSH in full

**Data Analysis**

- Line 2: Correct the error in the acronym MADR’s from MDAR’s

**Results**

**Incidence of major adverse drug reaction**

- Line 1, Paragraph 2: Kindly take a second look at the sentence ‘The incidence rate of MADR among females and males was 4.2/4278 and 3/5638 person months’ in comparison with the female: male population in Table 1, Review and confirm that its collaborative and correct

**Discussion**

- Line 5: Should read…. The study reports that ….
- Second Paragraph, Line 2: Revise to read: ‘in Debre Markos (in Ethiopia) and Mali’.

**Conclusions**

- Revise the first sentence, especially the quotation of the value ‘3.5/1000’.

**Summary**

1. Manuscript requires minor revisions indicated.

Thanks
